# Comparative Study on Structural Differences in Monosaccharide Layers Using PLD and PED Techniques

**DOI:** 10.3390/molecules29215095

**Published:** 2024-10-28

**Authors:** Agata Niemczyk, Agata Goszczyńska, Dariusz Moszyński, Paweł Figiel, Sebastian Fryska, Jolanta Baranowska

**Affiliations:** 1 Department of Materials Technology, Faculty of Mechanical Engineering and Mechatronics, West Pomeranian University of Technology in Szczecin, Piastów Avenue 19, 70-310 Szczecin, Poland; 2 Department of Polymer and Biomaterials Science, Faculty of Chemical Technology and Engineering, West Pomeranian University of Technology in Szczecin, Piastów Avenue 42, 71-065 Szczecin, Poland; 3 Department of Chemical and Environment Engineering, Faculty of Chemical Technology and Engineering, West Pomeranian University of Technology in Szczecin, Pułaskiego 10, 70-322 Szczecin, Poland

**Keywords:** pulsed electron beam deposition, pulsed laser beam deposition, glucose layer, physical structure, chemical structure

## Abstract

To demonstrate the feasibility of obtaining low-molecular-weight organic films (below 200 Da) using non-solvent PVD processes, glucose layers were produced via pulsed laser deposition (PLD) and pulsed electron beam deposition (PED) methods. Glucose was chosen due to its fundamental role in various biological processes, and because this low-molecular-weight compound is a solid at room temperature, which is required for both techniques. The physical and chemical structures of the deposited glucose layers were characterized by optical, scanning electron, and atomic force microscopy, as well as by X-ray diffraction, X-ray photoelectron, and infrared spectroscopy. Both PLD and PED methods resulted in glucose layers with good chemical structure preservation (with minor oxidation observed in PED) while yielding films with distinct physical properties. This opens up the possibility of tailoring organic layers with specific characteristics depending on the application, by choosing the deposition method.

## 1. Introduction

Thin layers/coatings of organic compounds are applied in various fields, and have particular importance in medicine, where they are used as active surfaces in products such as drug-eluting stents or wound dressings [1,2]. They are also significant in applications like organic electronics and chemical sensors [3]. The physical and chemical structure, thickness, and topography of the layers impact their effectiveness and safety in medical applications, and strongly depend on the process method used. Practically, traditional application techniques, such as spin-coating, dip-coating, or spray-coating, rely on the dissolution of organic compounds, which has serious drawbacks. Residues from the solvents, even in trace amounts, can have a negative effect on living organisms or on other environments in contact with the layer. Using water as a solvent is an alternative; however, water presents challenges due to its low evaporation rate and the formation of hydrates in the form of bound water that cannot be removed by simple evaporation [4]. In addition, many of the organic compounds used are insoluble in water.

Physical vapor deposition (PVD) techniques include a series of vacuum deposition methods using high energy to vaporize source material and deposit it on the surface. All types of materials, i.e., metals, ceramics, glass, and polymers, can be used to obtain solvent-free thin coatings with a precise thickness. Historically, the focus of PVD technology has been on the deposition of metals and inorganic compounds; therefore, for those materials, PVD methods are the most advanced and applied [5]. However, for almost four decades, there has been a growing interest in using polymers, which was started by research with polytetrafluoroethylene (PTFE) [6]. PTFE is a thermoplastic polymer with a high melting point, very high melt viscosity, and insolubility in traditional organic solvents. These processing challenges were a trigger to utilize various PVD techniques to obtain PTFE coatings. The success of producing PTFE coatings achieved by methods such as vacuum evaporation, magnetron sputtering, or pulsed laser deposition (PLD) [7,8,9,10] led to attempts with other types of polymers. However, the very high energy used in these techniques leads to polymer chain scission, with various (often low) chain reconstruction in the deposited coating. Therefore, the primary challenge faced by PVD methods has been minimizing the degradation of polymeric materials when exposed to the high-energy beam. In tackling this issue, pulsed high energy beam deposition methods, i.e., pulsed laser deposition and pulsed electron beam deposition (PED), stand out as promising technologies, showcasing notable success in preserving the chemical structure of polymers [11]. Our previous studies have demonstrated that these two techniques allow the transfer of larger polymer chain fragments; thus, chain scission occurs to a smaller extent [12,13]. This capability is crucial for achieving the desired chemical and physical properties of the deposited layers. Success in depositing polymers depends on carefully balancing the energy input. It should be high enough to pass the ablation threshold, but still as low as possible to avoid destroying the covalent bonds within the polymer. Overcoming van der Waals forces occurring between macromolecules in a polymer is relatively straightforward compared to overcoming the strong ionic interactions between small molecules in ceramics. Nevertheless, for a polymer, the ablation threshold also arises from the energy required to transport the entire macromolecule, desirably without its degradation, which usually remains a problem. Hypothetically, if low-molecular-weight organic compounds are used, the transport of a molecule, or, more precisely, the energy required to induce it during the PLD or PED process, should be much lower. Consequently, the creation of a layer of low-molecular-weight organic compounds by these non-solvent methods should be feasible and enable good chemical structure preservation.

The aim of our study was to explore the feasibility of depositing a low-molecular-weight organic compound (below 200 Da) using two PVD methods and to define the structural differences of the layers obtained. Glucose was selected as the model compound due to its presence in nature and its critical role in various biological processes, for example, serving as immune regulatory materials [14], making it an ideal candidate to investigate the potential of PLD and PED in organic material deposition. It is also important that the low-molecular-weight compound is a solid at room temperature, which is required for both techniques. The physical and chemical structures of the deposited glucose were analyzed using optical, scanning electron, and atomic force microscopy, as well as X-ray diffraction, X-ray photoelectron, and infrared spectroscopy.

## 2. Results and Discussion

### 2.1. Physical Structure of Glucose Layers

**Figure 1:**
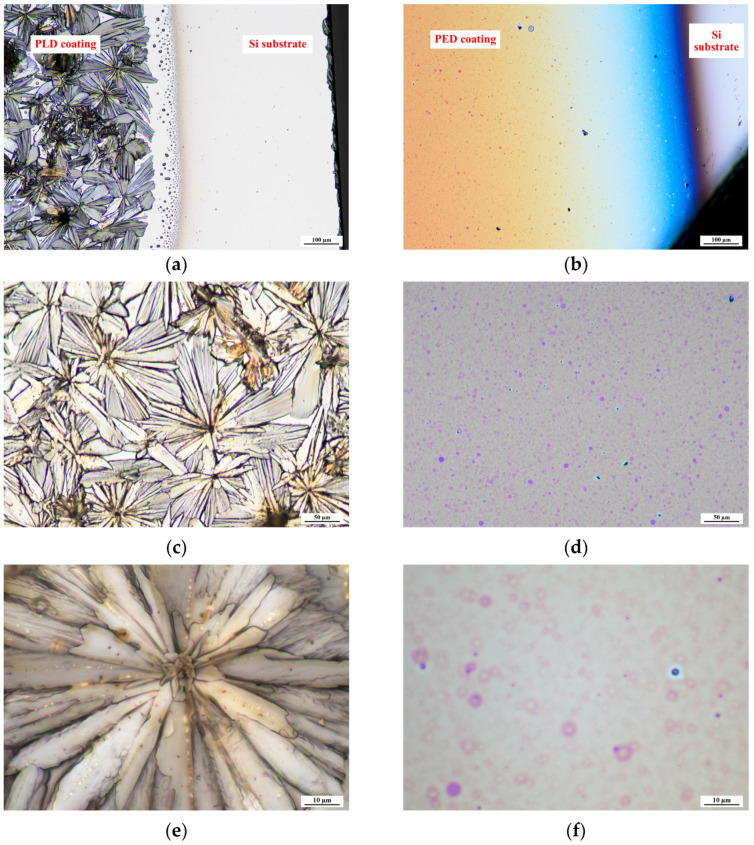
Optical micrographs of GluPLD (**a**,**c**,**e**) and GluPED (**b**,**d**,**f**). The micrographs scale bars are as following (**a**,**b**) = 100 µm, (**c**,**d**) = 50 µm, and (**e**,**f**) = 10 µm.

Glucose was deposited on a Si wafer using two PVD techniques, namely, pulsed laser deposition (sample signature GluPLD) and pulsed electron beam deposition (sample signature GluPED). Figure 1 shows micrographs obtained by optical microscopy of both layers with various magnifications. The GluPLD layer is characterized by a crystalline structure, which corresponds to the typical structure of glucose, which is a crystalline compound [15,16]. On the surface, clearly developed crystallites are visible with flower-like shapes. Their form developed in all three planes, including the z plane. The GluPED layer is completely different in morphology. The surface is as flat as the substrate and has no visible crystallites. However, round drops are present on the surface with an inter-droplet spacing of approximately 9 microns. It has been shown previously that polymer coatings obtained by the PED method are often characterized by a reduced degree of crystallinity, or even a rubber-like behavior. However, in the coatings referred to in [17], the reason for the increased amorphousness of the coating was the branching and spatial cross-linking of the long polymer chains, which, in the case of glucose, is highly unlikely. Additionally, glucose has a very strong tendency to crystallize [18]. Thus, the lack of a microcrystalline structure is rather unexpected and difficult to explain at this stage of the research.

**Figure 2:**
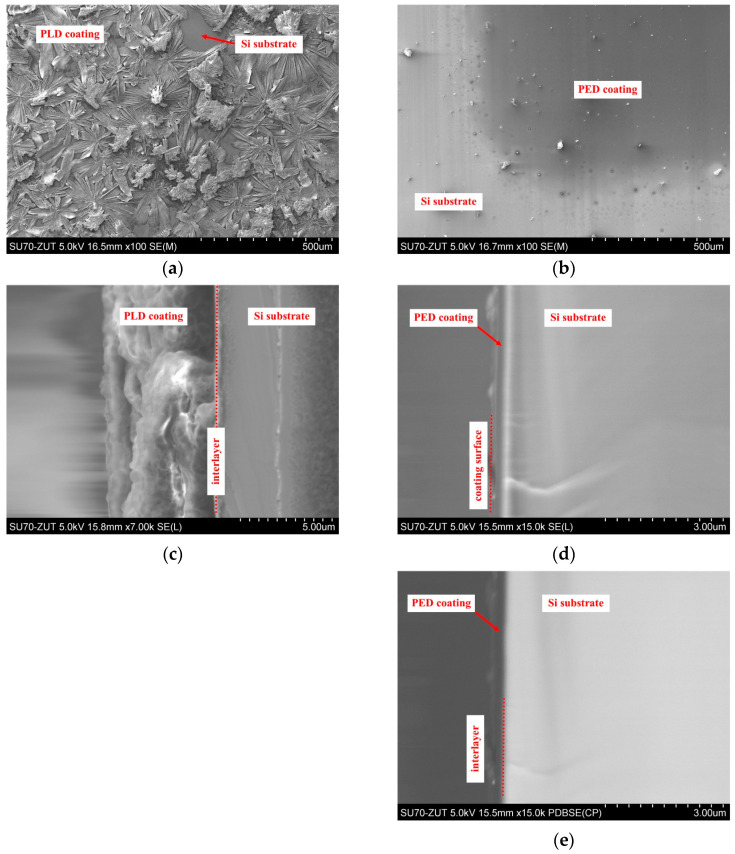
SEM images of GluPLD and GluPED layers: top surface of GluPLD (**a**) and GluPED (**b**); cross-section of the layer of GluPLD (**c**) and GluPED (**d**,**e**). The scale bars are as follows: (**a**,**b**) = 500 µm, (**c**) = 5 µm, and (**d**,**e**) = 3 µm.

Figure 2 presents SEM images of the surface and cross-section of the deposited GluPLD and GluPED layers. Consistent with the optical micrographs, the GluPLD layer produced by the PLD method comprises flower-shaped crystallites (Figure 2a), with a film thickness of approximately 6 μm (Figure 2c). Notably, the GluPLD layer exhibits numerous uncovered areas between the crystallites, resulting in a discontinuous morphology (as corroborated in Figure S1 in the Supplementary Materials (SM) through a comparison of SE and BSE mode images). In contrast, the GluPED is continuous, uniformly covering the substrate with a significantly lower thickness of approximately 350 nm (Figure 2d,e).

**Figure 3:**
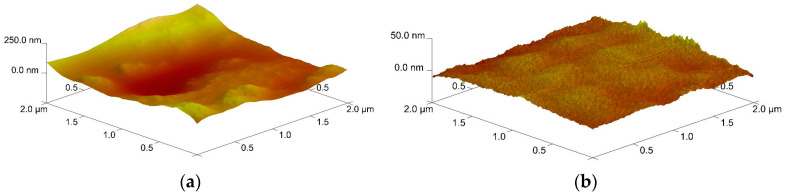
Topography of (**a**) GluPLD and (**b**) GluPED layers; AFM.

The surface topography of the GluPLD and GluPED layers was characterized using AFM, with the resulting 3D images shown in Figure 3. Due to the large height variation among the crystallites constituting the GluPLD layer (exhibiting microscale roughness), AFM measurements were only feasible on the flat regions of the “petals” of the flower-shaped crystallites. Even so, height variations reached 250 nm within the 2 × 2 μm region selection. In contrast, the GluPED layer exhibits much smaller height variations, on the order of tens of nanometers. Interestingly, this layer displays a uniform topography across its entire surface. It is notable that the morphology and topography of both glucose layers are significantly different, even though PLD and PED techniques are often regarded as being quite similar. Typically, both the inorganic and polymer layers produced by these methods exhibit a number of similarities in their physical structure; differences between the techniques are primarily observed in the preservation of the chemical structure of the initial polymer material. Here, in the case of a low-molecular-weight compound, there are clear significant differences in the physical structure of the layers.

**Figure 4:**
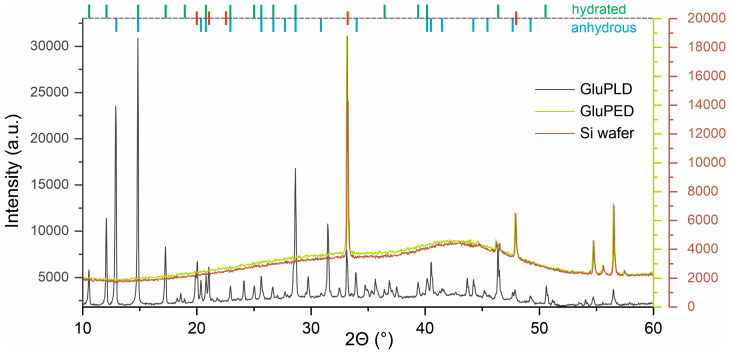

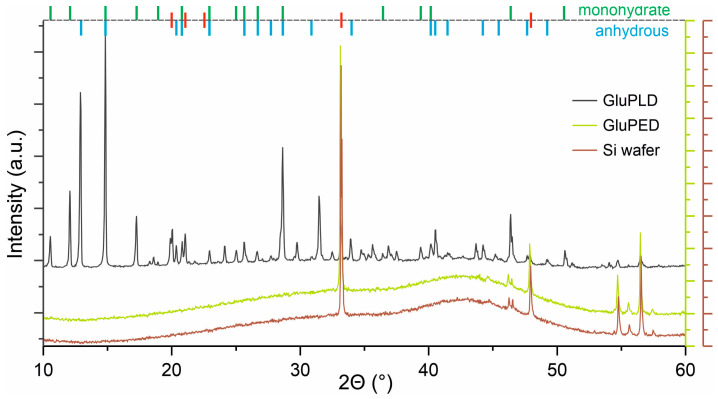
X-ray diffraction patterns for GluPLD, GluPED, and bare Si wafer. The diffraction peaks characteristic of glucose monohydrate are indicated by green lines, and those of anhydrous glucose by blue lines. New reflections are marked with red lines.

To complete the evaluation of the physical structure of the GluPLD and GluPED layers, XRD analysis was performed. The glucose used for layer deposition (target material) was a mixture of anhydrous and hydrated α-glucose, as confirmed by diffraction studies (Figure S2, in the SM). The diffraction pattern obtained for the GluPLD coating showed the presence of crystalline structures (Figure 4). The positions of the identified reflections in the diffractogram obtained for the GluPLD layer coincided largely with the reflections of the reference glucose. However, variations in the intensity ratios of some reflections were observed. Additionally, four extra reflections appeared at 2Θ = 20.0°, 21.0°, 24.1°, and 46.4°, which are generally absent in the diffraction patterns of any form of glucose, whether in the α or β anomer [15,16,19]. An additional reflection, at 2Θ = 33.1° overlapped with the Si substrate peak. The differences in the relative intensities of the diffraction reflections compared to the starting material could suggest the presence of texture in the layers, likely due to substrate interactions, which may induce the preferential crystallization of the material in certain directions during deposition.

The XRD spectrum of the GluPED layers was nearly identical to that of the Si substrate, which is largely due to the small thickness of the deposited layer. However, if the layers exhibited significant crystallinity, as seen for the GluPLD layer, one would expect to detect at least weak outlines of the most intense reflections, which is not the case for the obtained layers. This would suggest a high degree of amorphization in the GluPED layers, which is consistent with the surface morphology observed via microscopic techniques, where the smooth, amorphous structure contrasts with the highly crystalline surface of the GluPLD layers.

### 2.2. Chemical Structure of Glucose Layers

**Figure 5:**
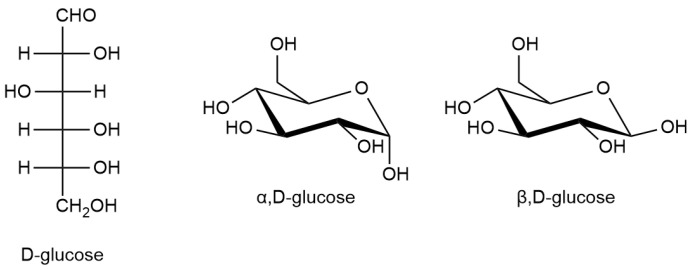
Structure of D-glucose (linear form) and α- and β-D-glucopyranose (cyclic anomers).

The chemical composition of glucose is relatively simple, consisting of carbon, hydrogen, and oxygen, with the molecular formula C_6_H_12_O_6_. However, the structural complexity of glucose is significant, as it can exist in various spatial configurations, including linear and cyclic forms. The cyclic form, in particular, has two anomers (α and β), which differ in the orientation of the OH group at the hemiacetal carbon atom relative to the plane of the ring (Figure 5), and form different crystal structures, as already mentioned.

The chemical composition of the layers obtained by PED and PLD techniques was analyzed using X-ray photoelectron spectroscopy (XPS). Carbon and oxygen atoms were identified as the main constituents on the surfaces of both samples. The XPS C 1s spectra of the samples are presented in Figure S3 (in the SM). The envelopes of the C 1s spectra of GluPLD and GluPED layers are alike, indicating a very similar chemical composition of both layers’ surfaces. Deconvolution of the experimental data was performed assuming four basic components of the C 1s transition. A reference component denoted as CH_x_, set to a binding energy of 285.0 eV, is attributed to all non-functionalized carbon atoms, in sp^3^ hybridization, bonded either with a second carbon atom or with hydrogen atoms. The component denoted as COH, shifted 1.5 eV from component CH_x_ in the direction of increasing binding energy, is ascribed to a group of carbon atoms linked to one atom of oxygen. The component denoted as C=O, shifted 2.8 eV from component CH_x_ in the direction of increasing binding energy, corresponds to a carbonyl functional group. The component denoted as COO, shifted 4.0 eV from component CH_x_ in the direction of increasing binding energy, is ascribed to a group of carbon atoms linked to two atoms of oxygen as in O–C=O moieties. The numerical values derived after applying the above model to all considered XPS C 1s spectra are listed in Table 1.

The component COH dominates the intensity in the spectrum of both samples. This observation is consistent with expectations for a spectrum of glucose, in which the main functional groups are C-OH groups. In addition, there is a significant C=O component, which may correspond to either the O-C-O functional groups also present in the glucose structure, or the carbonyl groups, C=O. There are no carbon atoms in the glucose structure that do not have a direct bond to an oxygen atom. Therefore, the CH_x_ component observed in the C 1s spectrum may be attributed to surface contamination by “adventitious carbon” [20], or possibly to carbon atoms derived from glucose and subject to reduction during deposition processes. The component COO is attributed to the presence of carbon atoms that originate from glucose undergoing partial decomposition and subsequent oxidation during deposition.

Comparing the XPS C 1s spectra and the calculated values of the fraction of the components of this spectrum after deconvolution, it can be concluded that the GluPLD layer shows a lower content of carbon–oxygen bonds on the surface than the GluPED layer.

**Figure 6:**
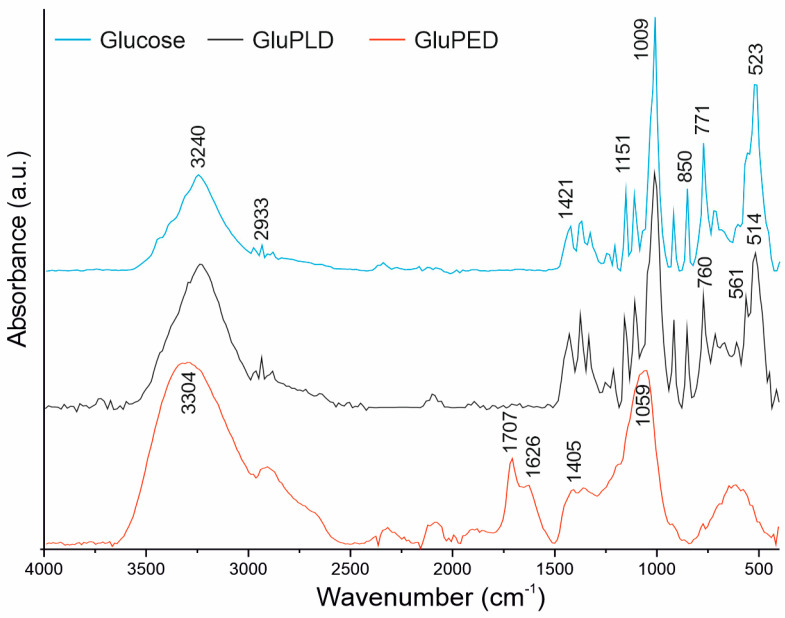

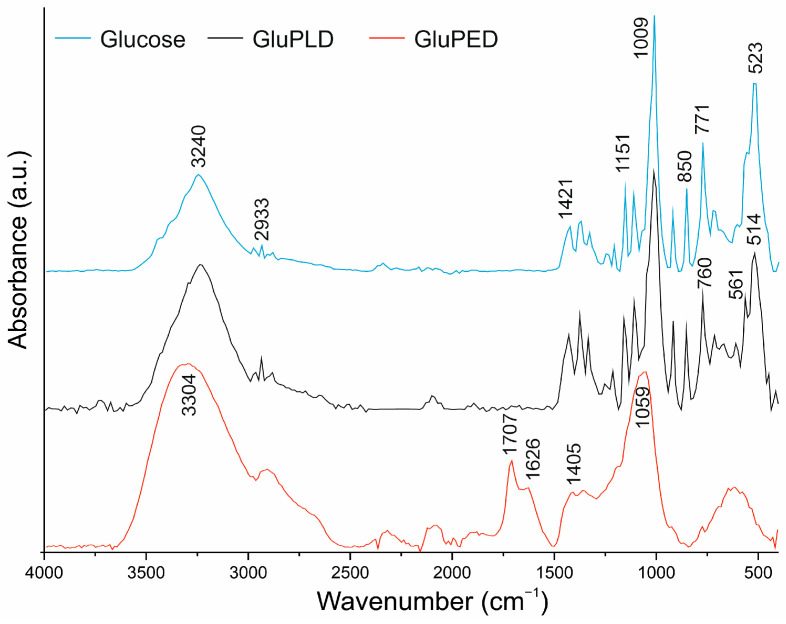
FTIR spectra of the GluPLD, GluPED, and referenced glucose.

Figure 6 presents the IR spectra of the glucose layers compared to the spectrum of the initial glucose material. The IR spectrum of the reference glucose has all typical absorption bands of the anomer mixture, in accordance with the literature [21,22]. The IR spectrum for the GluPLD layer shows the presence of all absorption bands found in the reference glucose spectrum, indicating that the PLD technique has resulted in the complete preservation of the glucose chemical structure in the layer. The only minor differences observed are slight variations in the intensity of absorption bands and some changes in the spectral profile below 850 cm⁻¹. Bands in this region are strongly influenced by the physical structure of glucose, particularly the degree of crystallinity and the type of glucose anomer (α or β). Therefore, these spectral differences likely stem from alterations in the physical, rather than chemical, structure. This finding is consistent with the XRD observations, which also revealed some differences between the reference glucose and the GluPLD layer.

In contrast, the IR spectrum for the GluPED layer shows significantly broader IR bands, affecting spectrum quality, which may be due to the low thickness of the coating, or the amorphous nature of the GluPED layer. Broadening of the IR absorption bands is typically observed in saccharides with a reduced degree of crystallization [23,24]. This is particularly evident in the OH absorption band in the range of 3600–3000 cm^−1^ and the C–O absorption band in the range of 1100–1000 cm⁻¹, where the peak maxima shift toward higher wavenumbers. This shift, referred to as a blue shift, likely results from the disordered structure of the amorphous GluPED layer, which prevents the formation of intermolecular hydrogen bonds that are commonly present in the crystalline phase of glucose.

Additionally, two new absorption bands are present in the spectrum of GluPED, at wavenumbers 1707 and 1626 cm^−1^. The band at 1707 cm⁻¹ corresponds to the presence of a carboxyl group suggesting glucose oxidation, which is consistent with the XPS analysis. This may be attributed to oxidation of the aldehyde group to a carboxyl group. The literature shows the examples in which glucose molecules undergo such an oxidation reaction under the influence of physical factors such as cold atmospheric plasma [25]. Therefore, we assume here that the exposure of the glucose molecule to the electron beam resulted in the partial conversion of glucose into glucuronic acid (or a similar derivative) during the PED process. For the initiation of the reaction, a hydroxyl or oxygen radical is required. Bonded water, present in hydrated glucose molecules (target material) could, under electron beam action, transform to such radicals. This means that the source of the significant differences between GluPLD and GluPED may originate from the bound water in the target material. Previous research conducted with protein compounds deposited by the PLD process postulates that, in the case of laser ablation, the presence of “trapped” water (or hydrated target material) has a positive effect on the preservation of the structure of the starting material. This is because of the absorption of a part of the laser energy, as in matrix-assisted pulsed laser evaporation [26]. In the case of pulsed electron beam deposition, the presence of water molecules acts as the source of free radicals, which can lead to the partial oxidation of the starting material. Also, as in the case of glucose, in addition to changing the chemical structure, this can lead to changes in the physical structure of the deposited material. This can be considered an advantage or disadvantage of the process, depending on the intended application.

The second new absorption band of the GluPED, at the wavenumber 1626 cm^−1^ is characteristic of hydrated saccharides [27] and indicates a significant amount of water molecules in the GluPED layer. The presence of water might partially explain the unexpected amorphous nature of the GluPED layer, since water molecules act as a plasticizer for saccharides [28].

Both PLD and PED techniques produced glucose layers with good preservation of the chemical structure, though the PED technique resulted in some oxidation. The techniques also led to glucose layers with significantly different physical structures, presenting opportunities to create organic layers with varying characteristics based on the intended application. The more efficient PLD process resulted in a thicker, highly crystalline and rough GluPLD layer, although it lacked continuity with some exposed substrate regions. In contrast, the PED process produced dense thin films, showing uniform morphology over the entire surface, but with less preservation of the chemical structure compared to the PLD method and with a distinctly amorphous appearance. To confirm the latter, an additional process was performed with a six-fold higher pulse number. The resulting thicker film with the same chemical structure as the thinner one was completely amorphous, as confirmed by XRD studies where only an amorphous halo was visible without any additional diffraction peaks (Figure S4). These findings suggest that each technique offers distinct advantages, enabling the production of organic layers with tailored properties for specific uses.

## 3. Materials and Methods

Glucose layers were deposited by means of a PED/PLD system (NEOCERA, Inc., Beltsville, MD, USA). Anhydrate D-(+)-Glucose powder (Chempur, Piekary Śląskie, Poland) was used to prepare the target (a disk of 50 mm diameter and ~5 mm height) by cold pressing with the addition of about ~100 µL of deionized water. Before the process, the target was dried at room temperature under reduced pressure (≤10^−4^ Torr). The same pressure (≤10^−4^ Torr) was maintained in the chamber overnight prior to the process. Glucose was deposited on silicon (100) substrates 10 × 10 mm in size, which, prior to deposition, was sonically cleaned in acetone, rinsed in acetone and isopropyl alcohol, and dried in a compressed air flow. In the case of the PLD method, an excimer laser (Coherent CompexPro 201F; He/Ne; KrF, λ = 248 nm, Santa Clara, CA, USA) with 20 ns pulse duration was used. For ablation, 3500 laser pulses with energy of 85 mJ were applied. The background argon gas pressure was 3 mTorr. In the case of the PED method, a pulsed electron beam source (PEBS) was used, operating at a voltage of 10 kV, with a background argon gas pressure of 8 mTorr Ar. The pulse width was 100 ns. Ablation was carried out using 10,000 and 60,000 electron beam pulses. For both techniques, the pulse repetition rate was 5 Hz and the distance between the target and substrate was 80 mm.

The layers were examined using light optical microscopy using a Nikon Ephiphot 200 (Nikon Corporation, Tokyo, Japan) and scanning electron microscopy using an FE-SEM SU-70 (Field Emission Scanning Electron Microscopy) microscope (Hitachi, Naka, Japan). Both the surface of the layer and its cross-section were observed. The inter-droplet spacing was calculated as the average distance between a droplet and surrounding droplets using ImageJ bundled with 64-bit Java 8 software. Four randomly selected droplets were measured. For SEM, two types of images were recorded: an image in topographic SE (secondary electrons) mode and an image in phase contrast BSE (backscattered electrons) mode. The accelerating voltage was 5 kV. The topography of the layers was examined by atomic force microscopy using a Veeco NanoScope Iva (Veeco, Dornach Munich, Germany) on an area of 2 × 2 µm in non-contact mode and a resonance frequency of 56 kHz. Identification of the crystalline phase was carried out by X-ray diffraction (XRD) using PANanalytical PW3040/60 X’Pert Pro apparatus (Malvern Panalytical, Malvern, UK) equipped with Cu Ka radiation at 35 kV and 40 mA. The diffraction spectra were acquired in continuous scan mode, covering a 2Θ angle range from 10 to 60°, with a step size of 0.0334° and a scan speed of 0.0142°/s. The X-ray photoelectron spectra were obtained using Mg *Kα* (hν = 1253.7 eV) radiation using a Prevac (Rogów, Poland) system equipped with a Scienta SES 2002 electron energy analyzer operating at constant transmission energy (E_p_ = 50 eV). The samples were attached to a stainless steel sample holder. Charging effects were corrected by using the C 1s component, denoted as CH_x_, and set to 285.0 eV. Data processing involved background subtraction by means of an “S-type” integral profile and a curve-fitting procedure based on a least-squares method (software CasaXPS 2.3.16). Infrared spectra were obtained using attenuated total reflection Fourier infrared spectroscopy (ATR-FTIR; Tensor, Bruker, Billerica, MA, USA). Then, 128 scans at a resolution of 2 cm^−1^ were carried out for each layer and for the reference glucose. All spectra are presented in the wavenumber range of 4000–400 cm^−1^ after baseline correction.

## 4. Conclusions

Our research has demonstrated that PLD and PED techniques can successfully produce glucose layers while preserving their chemical structure and, in this way, opening up new possibilities for the solvent-free fabrication of low-molecular-weight organic films.

The PLD process showed high efficiency (deposition rate was 1.7 nm/laser pulse) and provided excellent preservation of the glucose chemical structure. However, it should be considered that water in the target could significantly contribute to this effect. The resulting GluPLD layer exhibited high crystallinity, although the crystal structure deviated slightly from that of the starting material. The crystallites in the layer contributed to microscopic roughness and discontinuities, with gaps of deposited glucose between them.

In contrast, the PED process, while less efficient (deposition rate 0.03 nm/electron beam pulse), produced a more uniform and continuous glucose layer with no signs of microcrystallinity. However, the preservation of the glucose chemical structure was poorer, with some level of oxidation. Again, this could be attributed to the presence of water in the starting material, which seems to offer less protection in the PED process than in the PLD process.

These results suggest that each technique offers distinct advantages, allowing for the production of organic layers with tailored features depending on the specific application.

## Figures and Tables

**Table 1:** Quantitative decomposition of XPS C 1s line of GluPLD and GluPED layers.

Sample	CH_x_	COH	C=O	COO	CH_x_	COH	C=O	COO
	Peak Position (BE in eV) of C 1s Components; in Parentheses FWHM in eV				% of Total Intensity of C 1s Components			
GluPLD	285.0 (1.5)	286.5 (1.3)	287.8 (1.6)	289.2 (1.3)	30	46	21	3
GluPED	285.0 (1.6)	286.5 (1.4)	287.8 (1.4)	289.1 (1.6)	32	41	18	7

## Data Availability

The data presented in this study are available upon request from the corresponding authors due to the fact that the data are a part of ongoing research. As such, controlled access to the data ensures that they are not prematurely disclosed or misinterpreted before additional analyses or publications are completed. We are committed to transparency and scientific rigor and welcome inquiries regarding the data. Please contact the corresponding author for further information or access to the data.

## Supplementary Materials

The following supporting information can be downloaded at https://www.mdpi.com/article/10.3390/molecules29215095/s1: Figure S1: SEM of GluPLD; Figure S2: XRD of the glucose target; Figure S3: X-ray photoelectron spectroscopy C 1 s spectra for GluPED and GluPLD layers; Figure S4: XRD of the GluPED (here thin GluPED) and glucose layer deposited with six-fold higher pulse number (here thick GluPED). The rectangle shows the infrared spectra of both layers.
