# Supplementary material for: Comparative Study on Structural Differences in Monosaccharide Layers Using PLD and PED Techniques
Source: Molecules. 2024 Oct 28;29(21):5095. doi: 10.3390/molecules29215095 (PMC11547590; doi:10.3390/molecules29215095)
Supplement: Supplementary file 1 [file molecules-29-05095-s001.zip › molecules-3220292-supplementary.pdf]

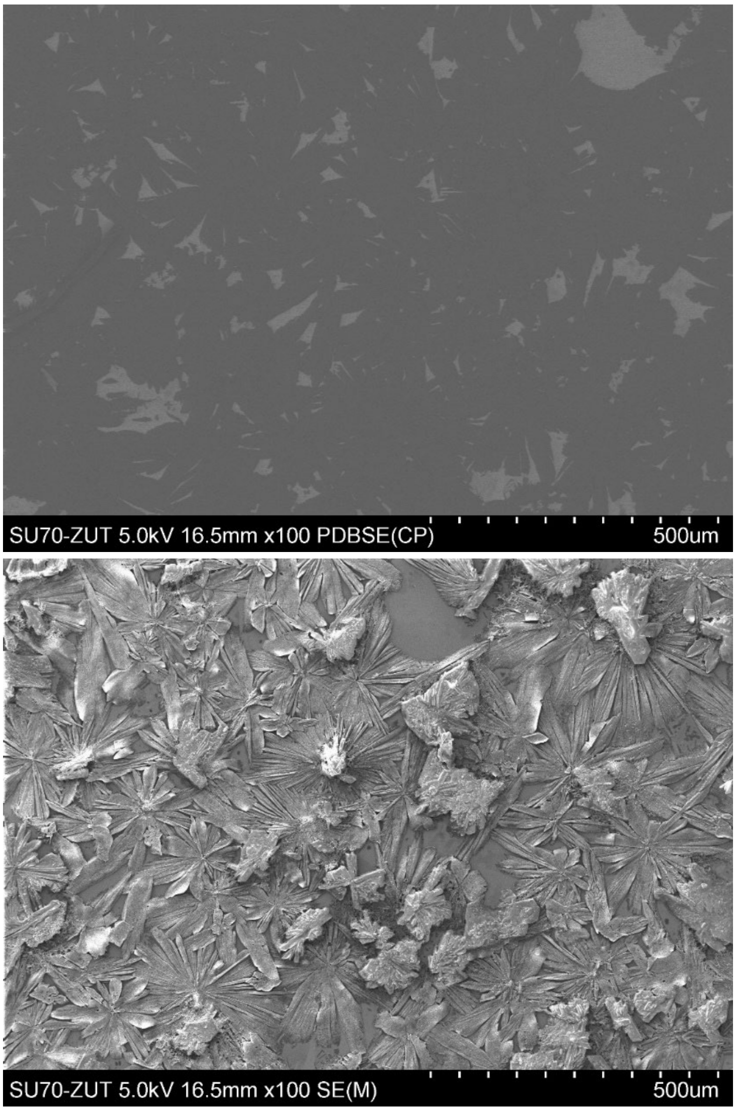

Figure S1. SEM of GluPLD

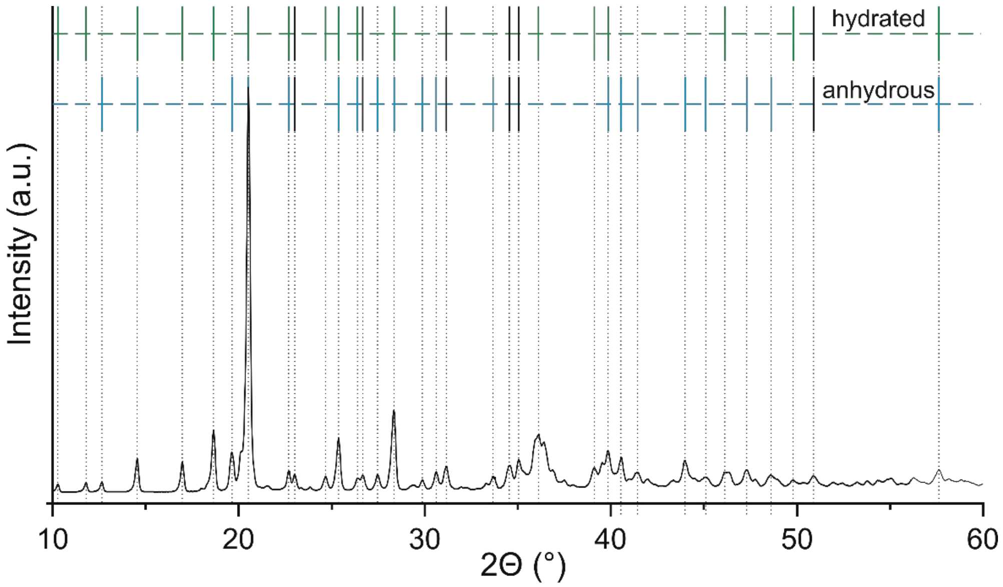

Figure S2. XRD of the glucose target. The diffraction peaks characteristic of glucose monohydrate are indicated by green lines, and those of anhydrous glucose by blue lines. Additional reflections are marked with black lines.

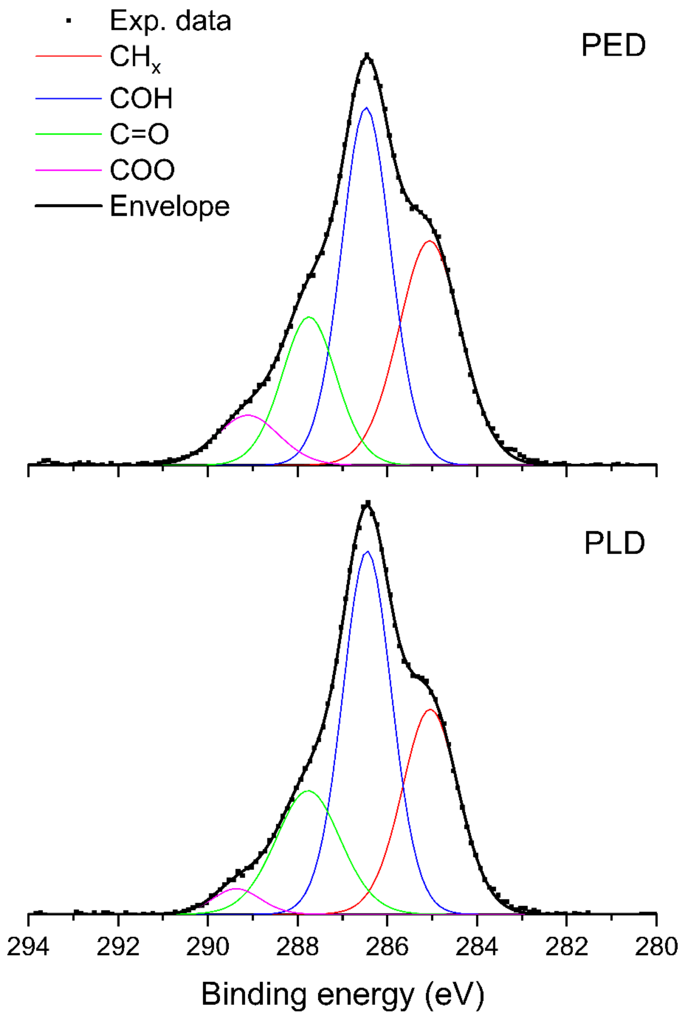

Figure S3. X-ray photoelectron spectroscopy C 1 s spectra for GluPED and GluPLD layers

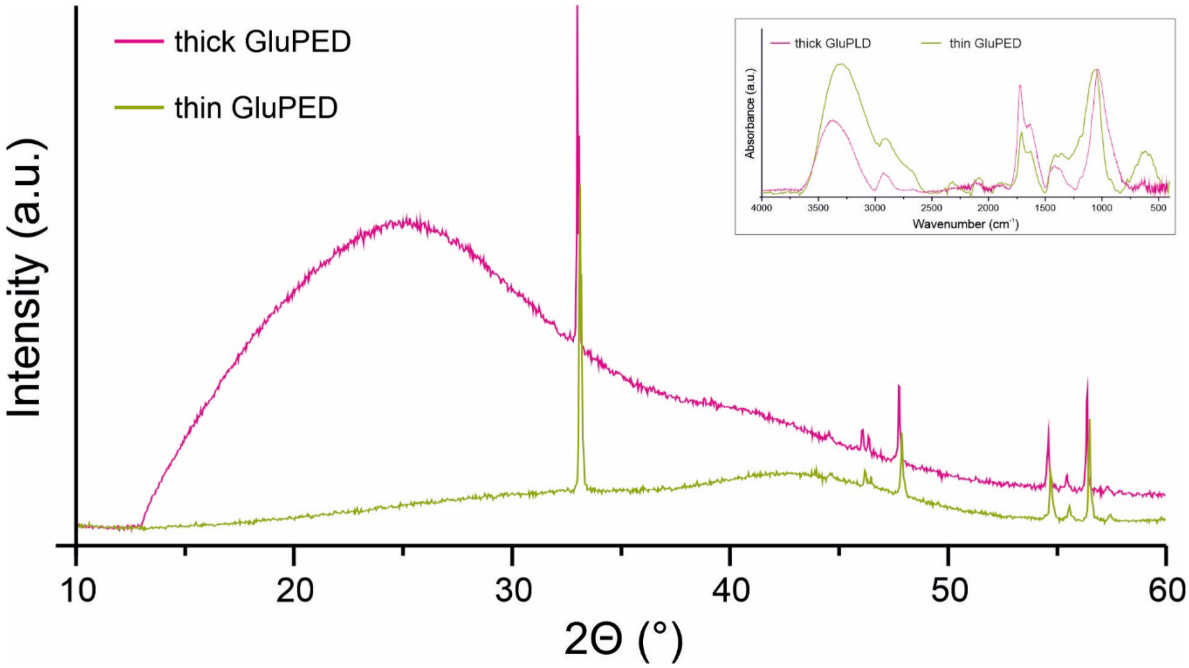

Figure S4. XRD of the GluPED (here thin GluPED) and glucose layer deposited with six-fold higher pulse number (here thick GluPED). The rectangle shows the infrared spectra of both layers.
